# Efficacy and safety of a novel ClearHemograsper for gastric endoscopic submucosal dissection: A prospective randomized controlled trial

**DOI:** 10.1038/s41598-026-41778-8

**Published:** 2026-03-11

**Authors:** Jin Hee Noh, Do Hoon Kim, Hee Kyong Na, Ji Yong Ahn, Jeong Hoon Lee, Kee Wook Jung, Kee Don Choi, Ho June Song, Gin Hyug Lee, Hwoon-Yong Jung

**Affiliations:** Department of Gastroenterology, Asan Medical Center, University of Ulsan College of Medicine, 88 Olympic-ro 43-gil, Songpa-gu, Seoul, 05505 Korea

**Keywords:** Endoscopic submucosal dissection, Gastric neoplasms, Hemostatic Forceps, Cancer, Diseases, Gastroenterology, Medical research, Oncology

## Abstract

Endoscopic submucosal dissection (ESD) has become a widely performed procedure for the management of gastric neoplasms. Effective hemostasis during ESD is critical for procedural success and patient safety. This study aimed to evaluate the efficacy and safety of a newly developed hemostatic device, ClearHemograsper, in gastric ESD. Patients who underwent ESD for gastric neoplasms between December 2022 and November 2023 were enrolled and randomly assigned to either the ClearHemograsper group or the Coagrasper group. We conducted a comprehensive analysis of clinicopathologic features and endoscopic treatment outcomes. A total of 157 patients, comprising 80 patients in the ClearHemograsper group and 77 patients in the Coagrasper group, were included in the final analysis. Analyses found no statistically significant differences in the bleeding control time (3.2 ± 2.9 vs. 3.5 ± 2.7 min, 95% Confidence interval, -1.227 to 0.526; *p* = 0.430) and total procedure time (15.2 ± 11.8 vs. 15.5 ± 10.4 min, *p* = 0.846) between the groups. En bloc resection was successfully performed for all lesions in both groups. Adverse events, including early bleeding (10.0% vs. 3.9%) within 48 h post-procedure and delayed bleeding (0.0% vs. 1.3%), showed no significant differences between the two groups (*p* = 0.211). All adverse events were effectively managed through endoscopic hemostasis. Novel ClearHemograsper demonstrates comparable efficacy and safety to Coagrasper, with preliminary evidence suggesting non-inferior or potentially superior hemostatic performance. These results demonstrate the comparable performance of domestic products in the Korean market, where overseas products occupy the majority of the device market share.

## Introduction

Gastric cancer is the third most frequently diagnosed cancer and the fourth primary cause of cancer mortality in South Korea, according to the Global Cancer Statistics (GLOBOCAN 2022)^1^. The National Cancer Screening Program for Gastric Cancer has increased the early detection rate of gastric cancer from 54.6% in 2009 to 66.0% in 2021, leading to more patients being eligible for endoscopic resection^2^. Endoscopic submucosal dissection (ESD) is widely accepted as the standard treatment for gastric neoplasms, including early gastric cancer and gastric dysplasia.

Bleeding is a common complication of ESD, occurring immediately during the procedure in approximately 22.6% to 90.6% of cases, with delayed bleeding reported in approximately 5% of cases^3^. Adequate hemostasis during ESD is critical to ensuring the patient’s hemodynamic stability and providing a clear view for the endoscopist, affecting the success and complication rates of the procedure^4^. Hemostasis provides a relatively quick and easy approach for managing bleeding sites or exposed vessels, with contact methods showing higher success rates compared to non-contact methods^5^. Hemostasis using hemostatic forceps is classified as a contact method. While minor bleeding from small blood vessels can typically be controlled using the ESD knife tip, large blood vessels or those with pulsation require hemostasis with specialized forceps.

**Fig. 1:**
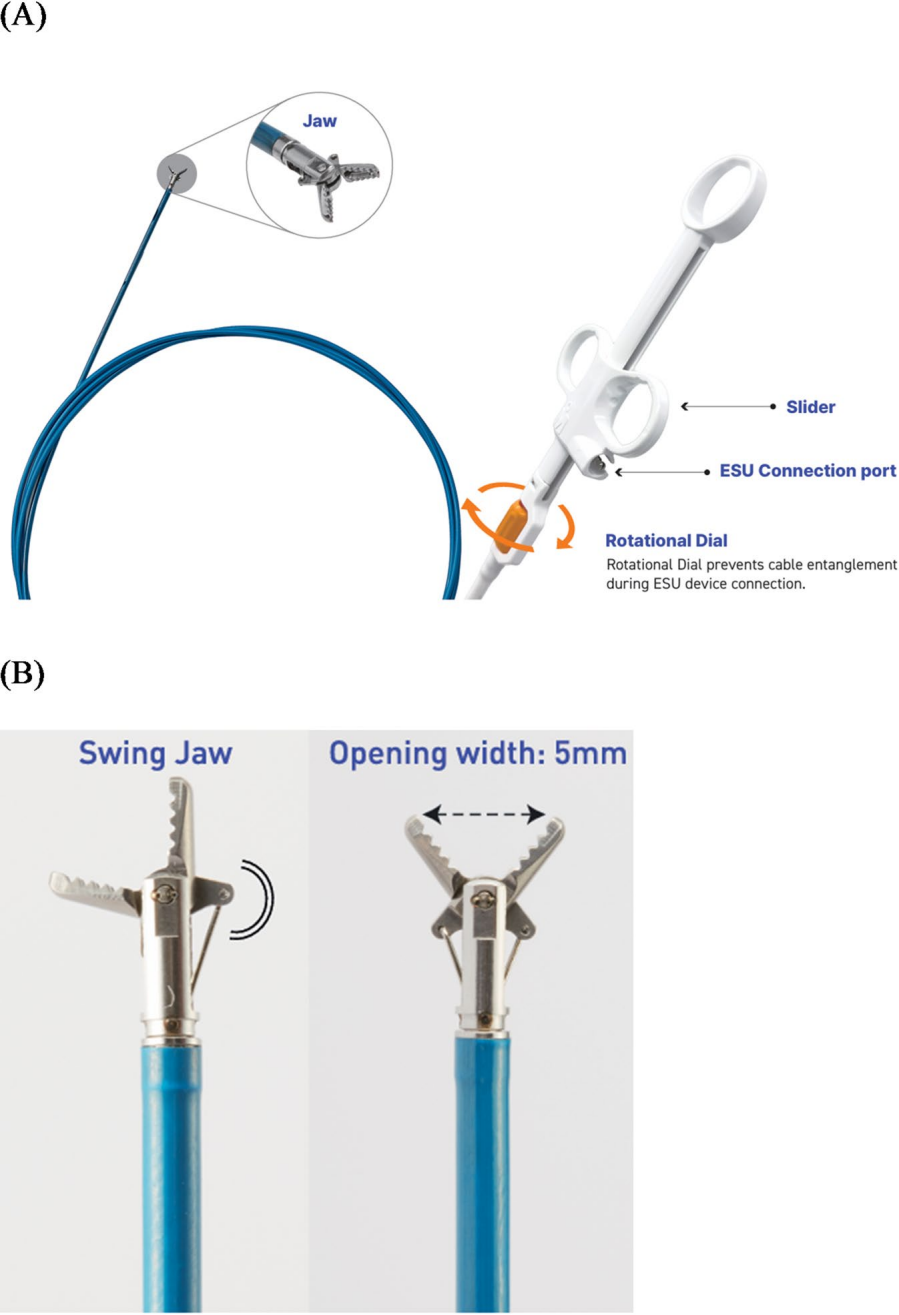
The ClearHemograsper. (**A**) Overall view of the forceps, showing both jaw and handle components, (**B**) Close-up image of the forceps head featuring a swing jaw with a maximum opening width of 5 mm. * Images courtesy of FineMedix Co., Ltd. (Seoul, Republic of Korea), reproduced with permission under CC BY 4.0 license.

The Coagrasper, a hemostatic forceps manufactured by Olympus Medical, has been used almost exclusively since its approval in Korea in 2017, with numerous studies demonstrating its hemostatic efficacy^6–8^. As part of Korea’s policy promoting domestically produced medical devices, FineMedix has developed a new hemostatic forceps called ClearHemograsper to provide a domestic alternative (Fig. 1); however, no studies have validated its efficacy and safety as of yet. Establishing evidence for the effectiveness and safety of high quality, cost-effective domestic products would enable their use not only in ESD, but also in other endoscopic procedures for the management of gastrointestinal bleeding. Therefore, this study aimed to evaluate the efficacy and safety of the newly developed ClearHemograsper compared to the established Coagrasper during gastric ESD procedures.

## Methods

### Study design and population

This single-center, prospective, single-blind, randomized controlled trial conducted at Asan Medical Center, a tertiary referral hospital in Seoul, Korea, enrolled patients aged 20–75 years with gastric neoplasms scheduled for ESD between December 6, 2022 and November 24, 2023. Patients with severe comorbidities, including decompensated liver cirrhosis, those undergoing hemodialysis or peritoneal dialysis, and those with hematologic disorders, such as thrombocytopenia < 50,000/µL were excluded. Additional exclusion criteria were suspected lymph node or distant metastasis before the procedure, previous history of endoscopic resection at the same site, history of gastrectomy, insufficient clinical information, and pregnancy or breastfeeding. Written informed consent was obtained from each patient before enrollment. Patients were randomly assigned in a 1:1 ratio to either the ClearHemograsper (FineMedix, Korea) group or the Coagrasper (Olympus, Japan) group according to a computer-generated random number table. The random sequence was stored in assignment tables, following allocation concealment before grouping. Patients were blinded to treatment group assignment, while the endoscopist and investigator were not blinded.

This study was approved by the Institutional Review Board of Asan Medical Center (IRB numbers: 2022 − 1459, approved November 2022) and registered with the Clinical Research Information Service (CRIS numbers: KCT0008165; registration date: February 9, 2023). Although patient enrollment began in December 2022, trial registration occurred in February 2023 due to administrative delays in finalizing funding contracts and institutional processes. The study protocol, including all endpoints, was predetermined and documented in the IRB-approved protocol before patient enrollment. The study adhered to the ethical standards outlined in the Declaration of Helsinki. This work was supported by ‘Supporting Project to evaluation Domestic Medical Devices in Hospitals’ funded by ‘Ministry of Health and Welfare (MOHW)’ and ‘Korea Health Industry Development Institute (KHIDI)’.

### Endoscopic procedures and definitions

All ESD procedures were performed by two experienced endoscopists (D.H.K., J.H.N.) according to standard techniques. The patients were hospitalized before the procedure according to the institution’s standard care protocols. After sedation, ESD was performed using a single-channel endoscope (GIF-HQ290; Olympus). When bleeding occurred during the procedure, hemostasis was performed using either the ClearHemograsper or Coagrasper, as determined by randomization. Patients underwent second-look endoscopy within 48 h of the procedure.

The time interval from initial mucosal injection to complete mucosal excision with successful hemostasis was measured as the total procedure time. The bleeding control time was defined as the total hemostasis time for all bleeding episodes during the procedure. Complete hemostasis was defined as the absence of visible blood flow and elimination of further bleeding risk, as assessed by the endoscopist. A procedure assistant participated in assessing this endpoint alongside the endoscopist. All patients were hospitalized with standardized monitoring including vital signs every 6 h and hemoglobin measurement on post-procedure day 1. Standardized second-look endoscopy was performed within 48 h for all patients. Follow-up was conducted between 2 weeks and 1 month with active symptom surveillance and laboratory tests. Bleeding events were graded as mild (minor oozing on endoscopy, hemodynamically stable, managed endoscopically), moderate (clinical symptoms requiring intervention but not transfusion), or severe (requiring transfusion, embolization, or surgery). The bleeding events after the procedure were categorized by their timing: early bleeding was characterized by the occurrence of hematemesis, hematochezia, or melena between procedure completion and second-look endoscopy, or by evidence of active or potential bleeding (Forrest classifications Ia, Ib, and IIa) during the second-look endoscopy. Delayed bleeding was defined as any bleeding manifestations, including hematemesis, melena, hematochezia, or significant hemoglobin reduction (≥ 2 g/dL) that occurred after the second-look endoscopy up until 1 month post-procedure^9^. Perforation was diagnosed endoscopically during the procedure, or radiographically when free air was revealed on chest imaging. Microperforation was defined as minimal free air on chest radiography in the absence of peritoneal irritation symptoms or signs.

### Outcomes

The primary outcome was the total time required for hemostasis of all bleeding episodes during ESD, measured in seconds. Secondary outcomes included the hemostasis success rate, total procedure time, and procedure-related complications. The clinical characteristics of patients and tumors, as well as endoscopic outcomes, including complete resection and curative resection were also analyzed.

### Sample size calculation

This clinical trial was designed as a non-inferiority study to compare the hemostatic performance of the ClearHemograsper with that of the Coagrasper. Based on previous prospective comparative pilot studies, the mean hemostasis time in the control group was 56.8 ± 51.8 s. Using a one-sided test with a significance level of 2.5%, statistical power of 80%, and a non-inferiority margin of 20 s, 113 subjects per group were required. Accounting for an anticipated dropout rate of 15%, the final target sample size was set at 266 patients (133 per group).

### Statistical analysis

Descriptive variables are summarized as mean and standard deviation. Differences in the patients and tumor characteristics between the two groups were appropriately compared using independent t-tests and chi-square or Fisher exact test, as appropriate. The Mann–Whitney U test was performed for nonparametric analysis of the two groups. A value of *p* < 0.05 was considered as statistically significant. All statistical analyses were performed using SPSS version 24 (IBM Corporation, Somers, NY, USA).

## Results

### Clinical characteristics of patients and tumors

**Fig. 2:**
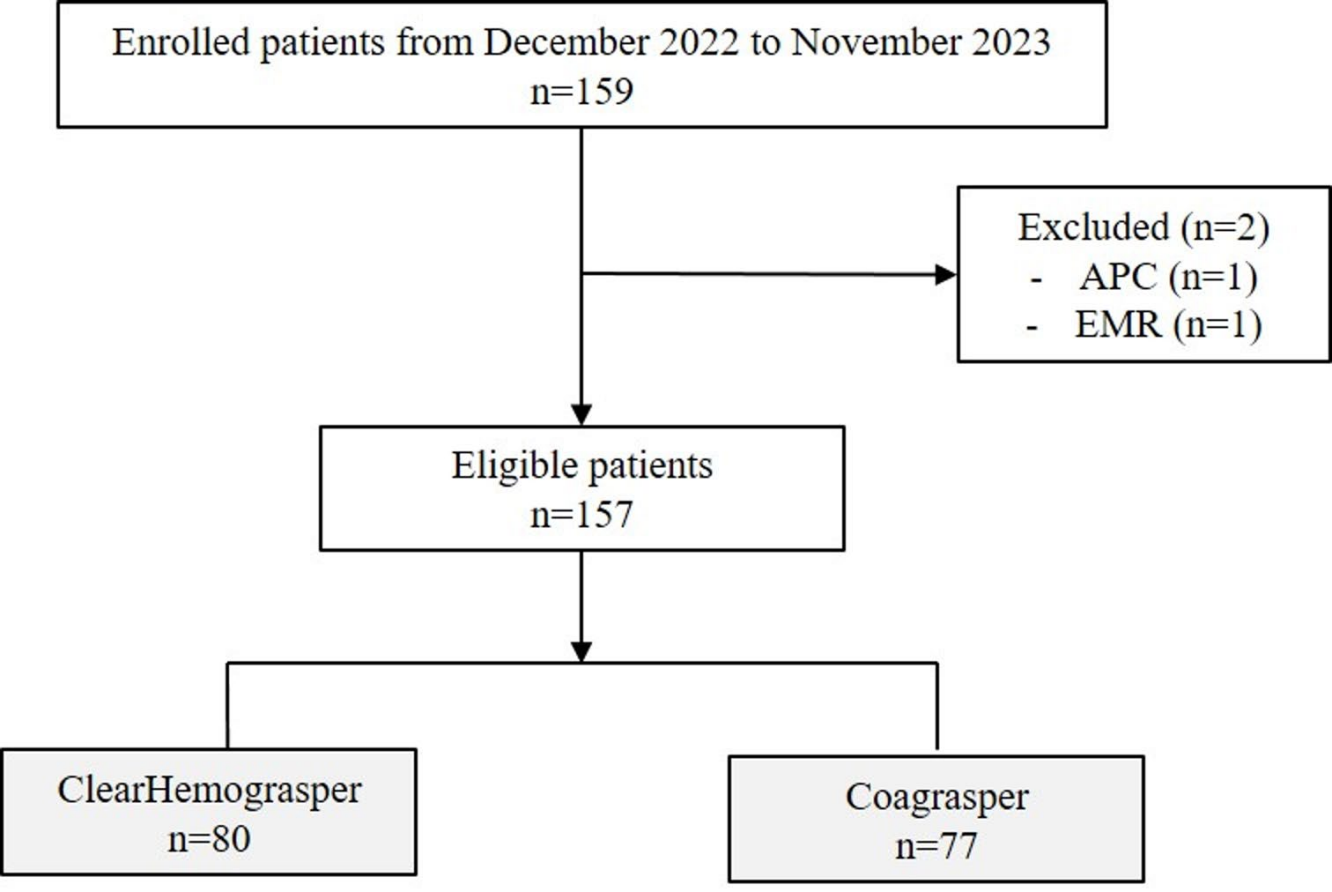
Flow chart of the enrolled patients and endoscopic treatment modality.

Overall, 159 patients were enrolled from December 2022 to November 2023. After excluding 2 patients (1 for argon plasma coagulation and 1 for endoscopic mucosal resection), 157 patients were included in the final analysis, with 80 patients allocated to the ClearHemograsper group and 77 to the Coagrasper group (Fig. 2).

Table 1 summarizes the demographic and clinical characteristics of the study population. The mean age was 63.1 ± 7.2 years, with men comprising 72.6%. Cardiovascular comorbidities were significantly more prevalent in the ClearHemograsper group compared to that of the Coagrasper group (13.8% vs. 3.9%, *p* = 0.047). No statistically significant differences were observed between the two groups regarding smoking status, alcohol consumption history, other comorbidities, ASA classification, or relevant medical history.

**Table 1:** Clinical characteristics of the study population.

Age, years	Total (*n* = 157)	ClearHemograsper (*n* = 80)	Coagrasper (*n* = 77)	*p*-value
	63.1 ± 7.2	63.5 ± 6.5	62.8 ± 7.9	0.539
Male (%)	114 (72.6)	56 (70.0)	58 (75.3)	0.479
Smoking				0.695
Non-smoker	69 (43.9)	37 (46.3)	32 (41.6)	
Ex-smoker	58 (36.9)	27 (33.8)	31 (40.3)	
Current smoker	30 (19.1)	16 (20.0)	14 (18.2)	
Alcohol				0.346
Non-user	60 (38.2)	35 (43.8)	25 (32.5)	
Ex-user	23 (14.6)	10 (12.5)	13 (16.9)	
Current user	74 (47.1)	35 (43.8)	39 (50.6)	
Comorbidities				
Hypertension	67 (42.7)	35 (43.8)	32 (41.6)	0.872
Diabetes	45 (28.7)	26 (32.5)	19 (24.7)	0.295
Chronic kidney disease	3 (1.9)	2 (2.5)	1 (1.3)	1.000
Liver cirrhosis	1 (0.6)	0 (0.0)	1 (1.3)	0.490
Cardiovascular disease^*^	14 (8.9)	11 (13.8)	3 (3.9)	0.047
Pulmonary disease	4 (2.5)	2 (2.5)	2 (2.6)	1.000
Malignancy	18 (11.5)	5 (6.3)	13 (16.9)	0.046
History of abdominal surgery	27 (17.4)	11 (13.8)	16 (21.3)	0.289
ASA classification				0.853
I	25 (15.9)	11 (13.8)	14 (18.2)	
II	116 (73.9)	61 (76.3)	55 (71.4)	
III	14 (8.9)	7 (8.8)	7 (9.1)	
IV	2 (1.3)	1 (1.3)	1 (1.3)	
Use of antithrombotic or anticoagulant agents	13 (8.3)	7 (8.8)	6 (7.8)	1.000

ASA, American Society of Anesthesiologists: I (a normal healthy patient), II (a patient with mild-to-moderate systemic disease who was well-controlled medically), and III (a patient with severe systemic disease with limited activity but not incapacitated).

^*^Cardiovascular diseases include ischemic heart disease, valvular heart disease, chronic heart failure, and arrhythmia.

Regarding the histopathological findings before procedure, early gastric cancer constituted 38.2% of all lesions, while gastric dysplasia accounted for the remaining 61.8% (Table 2). Additional endoscopic characteristics, including tumor location and gross morphological features, demonstrated no significant differences between the two groups.

**Table 2:** Endoscopic and histopathologic characteristics of tumors.

Location-1	Total (*n* = 157)	ClearHemograsper (*n* = 80)	Coagrasper (*n* = 77)	*p*-value
				0.137
Upper	20 (12.7)	6 (7.5)	14 (18.2)	
Middle	58 (36.9)	32 (40.0)	26 (33.8)	
Lower	79 (50.3)	42 (52.5)	37 (48.1)	
Location-2				0.944
Anterior wall	32 (20.4)	17 (21.3)	15 (19.5)	
Posterior wall	42 (26.8)	21 (26.3)	21 (27.3)	
Lesser curvature	51 (32.5)	27 (33.8)	24 (31.2)	
Greater curvature	32 (20.4)	15 (18.8)	17 (22.1)	
Histological diagnosis (pre)				0.227
Low-grade dysplasia	67 (42.7)	39 (48.8)	28 (36.4)	
High-grade dysplasia	30 (19.1)	12 (15.0)	18 (23.4)	
Gastric cancer	60 (38.2)	29 (36.3)	31 (40.3)	
Gross morphology				0.956
Flat-elevated	67 (42.9)	35 (43.8)	32 (42.1)	
Flat	31 (19.9)	15 (18.8)	16 (21.1)	
Flat-depressed	58 (37.2)	30 (37.5)	28 (36.8)	
Presence of ulcer	0 (0.0)	0 (0.0)	0 (0.0)	

### Efficacy outcomes

**Fig. 3:**
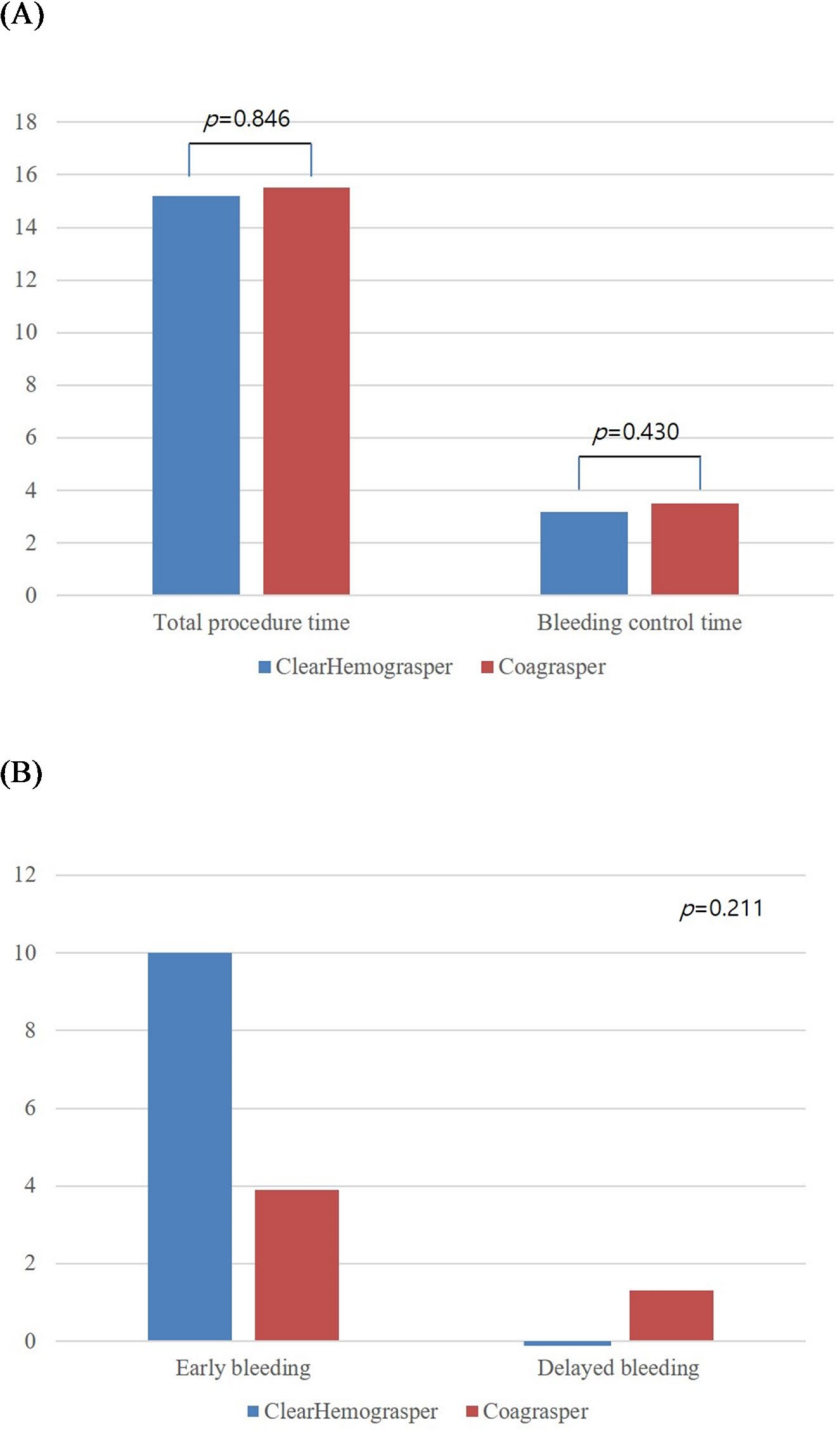
Comparison of clinical outcomes of procedure between the ClearHemograsper and Coagrasper groups. (A) Total procedure time (15.2 ± 11.8 vs. 15.5 ± 10.4 min, *p* = 0.846) and bleeding control time (3.2 ± 2.9 vs. 3.5 ± 2.7 min, *p* = 0.430), (B) Adverse events including early bleeding (10.0% vs. 3.9%) and delayed bleeding (0.0% vs. 1.3%, *p* = 0.211).

There were no statistically significant differences in the total procedure time (15.2 ± 11.8 vs. 15.5 ± 10.4 min, *p* = 0.846) between the ClearHemograsper and Coagrasper groups (Fig. 3A). Similarly, the bleeding control time was comparable between the two groups (3.2 ± 2.9 vs. 3.5 ± 2.7 min, 95% Confidence interval, −1.227 to 0.526; *p* = 0.430). Complete hemostasis was achieved for all lesions during the procedure. En bloc resection was successfully performed for all lesions in both groups (Table 3). The complete resection rate was 96.3% in the ClearHemograsper group and 94.8% in the Coagrasper group (*p* = 0.716), while the curative resection rate was 92.5% and 87.0%, respectively (*p* = 0.299).

**Table 3:** Clinical outcomes of endoscopic submucosal dissection for tumors.

Histological diagnosis (post)	Total (*n* = 157)	ClearHemograsper (*n* = 80)	Coagrasper (*n* = 77)	*p*-value
				0.342
Low-grade dysplasia	43 (27.4)	25 (31.3)	18 (23.4)	
High-grade dysplasia	20 (12.7)	11 (13.8)	9 (11.7)	
Gastric cancer	87 (55.4)	39 (48.8)	48 (62.3)	
Others (gastritis etc.)	7 (4.5)	5 (6.3)	2 (2.6)	
Depth of invasion				0.118
Mucosa	69 (79.3)	34 (87.2)	35 (72.9)	
Submucosa	18 (20.7)	5 (12.8)	13 (27.1)	
Tumor size, mm (*n* = 150)	15.8 ± 10.4	16.2 ± 11.9 (*n* = 75)	15.3 ± 8.8 (*n* = 75)	0.575
Resected specimen size, mm	34.8 ± 10.9	35.1 ± 11.1	34.6 ± 10.8	0.762
Lymphovascular invasion	0 (0.0)	0 (0.0)	0 (0.0)	1.000
Perineural invasion	0 (0.0)	0 (0.0)	0 (0.0)	1.000
Outcomes				
En bloc resection	157 (100.0)	80 (100.0)	77 (100.0)	1.000
Complete resection	150 (95.5)	77 (96.3)	73 (94.8)	0.716
Curative resection	141 (89.8)	74 (92.5)	67 (87.0)	0.299

Following ESD and comprehensive histopathological examination, the final diagnoses revealed a shift in distribution: the proportion of gastric dysplasia decreased to 40.1% while early gastric cancer increased to 55.4% (Table 3). Additionally, 4.5% of lesions previously diagnosed as low-grade dysplasia (*n* = 6) or gastric cancer (*n* = 1) were reclassified as non-neoplastic lesions, such as gastritis. There were no significant differences in mean tumor size (16.2 ± 11.9 vs. 15.3 ± 8.8 mm, *p* = 0.575) or resected specimen size (35.1 ± 11.1 vs. 34.6 ± 10.8 mm, *p* = 0.762) between the two groups. No lymphovascular or perineural invasion was observed in any of the lesions.

### Safety profile

Overall, there was no statistically significant difference in the bleeding complications between the two groups (*p* = 0.211). Early bleeding occurred in 10.0% of cases (*n* = 8) in the ClearHemograsper group compared to 3.9% (*n* = 3) in the Coagrasper group, as demonstrated in Fig. 3B. All bleeding events were detected during second-look endoscopy that were characterized as minor oozing. Delayed bleeding was observed in 0.0% of the ClearHemograsper group and 1.3% (*n* = 1) of the Coagrasper group. All bleeding events were successfully managed through endoscopic hemostasis without requiring surgical intervention.

## Discussion

This prospective randomized controlled trial is valuable in demonstrating the comparable efficacy and safety of a novel domestic hemostatic device, the ClearHemograsper, to the established Coagrasper during gastric ESD procedures, particularly in the Korean market, where overseas products occupy the majority of the endoscopic device market share. Our findings revealed comparable hemostatic performance between the two devices, with no significant differences in the bleeding control time (3.2 ± 2.9 vs. 3.5 ± 2.7 min) and total procedure time (15.2 ± 11.8 vs. 15.5 ± 10.4 min). Both devices achieved a 100% en bloc resection rate, with comparable rates of complete (96.3% vs. 94.8%) and curative resection (92.5% vs. 87.0%). The safety profiles were also comparable, with no statistically significant differences in early (10.0% vs. 3.9%) or delayed bleeding (0.0% vs. 1.3%) rates between the ClearHemograsper and Coagrasper groups, respectively.

Effective hemostasis during ESD is critical for both procedural success and patient safety, representing an important technical challenge that directly impacts the clinical outcomes^8,10^. Inadequate hemostasis can lead to extended procedure times, reduced visibility, and increased risks of adverse events, particularly bleeding complications^3,11,12^. The achievement and maintenance of clear endoscopic visualization through effective hemostasis are essential for accurate dissection along the appropriate plane, which directly correlates with successful en bloc resection rates and reduced perforation risk^13^. Furthermore, the preemptive management of visible vessels before and after dissection has been recognized as a key factor in preventing both immediate and delayed bleeding events.

Although various hemostatic forceps have been developed to control bleeding and enhance safety, their range is more limited compared to that of the ESD knife^14–18^. Furthermore, there is a lack of studies comparing the effectiveness of different hemostatic forceps, making it difficult to determine which type is most efficient during ESD. At present, the Coagrasper remains the predominant choice of hemostatic forceps for gastric ESD procedures^6,7^. In the present study, both hemostatic devices demonstrated comparable and high efficacy in achieving complete hemostasis during the procedure. These results align with previous studies on the Coagrasper, which reported successful hemostasis rates of 96%−100% in various gastrointestinal procedures^19^. The complete hemostasis success rate of 100% in both groups underscores the technical adequacy of the novel ClearHemograsper. A previous study reported a mean bleeding control time of 56.8 ± 51.8 s with the Coagrasper^8^, which is comparable to our findings when converted to seconds (210 ± 162 s). The slightly longer bleeding control time in our study may be attributed to the differences in case complexity, vessel size, and methodological distinctions. Particularly, our measurement included the cumulative time for all bleeding episodes throughout the entire procedure rather than individual bleeding events.

The safety profiles of both hemostatic forceps were comparable, with no significant differences in adverse events between groups. The overall early bleeding rate in our study (7.0%) is within the low range of previously reported rates of gastric ESD (5%−22%)^3^. Although the early bleeding rate was numerically higher in the ClearHemograsper group (10.0% vs. 3.9%), this difference did not reach statistical significance and may be related to the higher prevalence of cardiovascular disease in the ClearHemograsper group (13.8% vs. 3.9%, *p* = 0.047). Cardiovascular disease is a known risk factor for gastrointestinal bleeding due to the use or history of antiplatelet agents and/or anticoagulants with bleeding tendencies, and it is also a risk factor for bleeding complications related to ESD^20^. All bleeding events were minor and successfully managed with endoscopic hemostasis without requiring emergent surgical intervention. Furthermore, all events were characterized as minor oozing bleeding detected during second-look endoscopy rather than clinically significant bleeding requiring emergent embolization or surgical intervention, supporting the safety profile of both devices in a real-world clinical setting. The rate of delayed bleeding was low in both groups (0.0% vs. 1.3%), consistent with previous reports showing that delayed bleeding occurred in approximately 5% of gastric ESD cases^21,22^. Notably, no delayed bleeding occurred in the ClearHemograsper group, suggesting adequate vascular hemostatic function of the device.

This study has several limitations. The most significant limitation was the inability to enroll the planned sample size of 266 patients. Following completion of the funding contract and disbursement of research funds, we faced time constraints in recruiting the target number of patients within the study period. Consequently, the final analysis included 157 patients (80 in the ClearHemograsper group and 77 in the Coagrasper group), representing 59% of the target enrollment. This reduced sample size substantially decreased the statistical power from the planned 80%, limiting our ability to establish non-inferiority conclusively. Therefore, this study should be interpreted as providing preliminary rather than definitive evidence. Another limitation is the lack of detailed per-episode hemostasis data, including the number of bleeding events per patient and vessel size-specific hemostasis times, which could provide additional insights into device performance in future studies. Additional limitations include the single-center design, which may limit the generalizability of our results, and the relatively short follow-up period of 1 month, which might not detect rare late complications.

In conclusion, this exploratory randomized controlled trial provides preliminary evidence that the novel ClearHemograsper demonstrates comparable hemostatic performance for gastric ESD procedures to the established Coagrasper. These findings provide evidence supporting the use of domestically produced, high quality, and cost-effective medical devices in endoscopic procedures, which aligns with national healthcare policies promoting domestic alternatives. Further multicenter studies with larger sample sizes and longer follow-up periods are warranted to confirm these promising results and evaluate the long-term outcomes of patients treated with the ClearHemograsper.

## Data Availability

The datasets used and/or analysed during the current study available from the corresponding author on reasonable request.
